# Targeted Gene Expression Profile Reveals CDK4 as Therapeutic Target for Selected Patients With Adrenocortical Carcinoma

**DOI:** 10.3389/fendo.2020.00219

**Published:** 2020-04-16

**Authors:** Raimunde Liang, Isabel Weigand, Juliane Lippert, Stefan Kircher, Barbara Altieri, Sonja Steinhauer, Constanze Hantel, Simone Rost, Andreas Rosenwald, Matthias Kroiss, Martin Fassnacht, Silviu Sbiera, Cristina L. Ronchi

**Affiliations:** ^1^Division of Endocrinology and Diabetology, Department of Internal Medicine, University Hospital of Wuerzburg, Würzburg, Germany; ^2^Institute of Human Genetics, University of Wuerzburg, Würzburg, Germany; ^3^Institute of Pathology, University of Wuerzburg, Würzburg, Germany; ^4^Department of Clinical Medicine and Surgery, School of Medicine and Surgery, University of Naples Federico II, Naples, Italy; ^5^Department of Endocrinology, Diabetology and Clinical Nutrition, University Hospital Zurich, Zurich, Switzerland; ^6^Medizinische Klinik und Poliklinik III, University Hospital Carl Gustav Carus Dresden, Dresden, Germany; ^7^Comprehensive Cancer Center Mainfranken, University Hospital of Wuerzburg, Würzburg, Germany; ^8^Institute of Metabolism and System Research (IMSR), University of Birmingham, Birmingham, United Kingdom

**Keywords:** adrenocortical tumor, targeted therapy, palbociclib, CDK4, IGF1R

## Abstract

Adrenocortical carcinomas (ACC) are aggressive tumors with a heterogeneous prognosis and limited therapeutic options for advanced stages. This study aims to identify novel drug targets for a personalized treatment in ACC. RNA was isolated from 40 formalin-fixed paraffin-embedded ACC samples. We evaluated gene expression of 84 known cancer drug targets by reverse transcriptase quantitative real time-PCR and calculated fold change using 5 normal adrenal glands as reference (overexpression by fold change >2.0). The most promising candidate cyclin-dependent kinase 4 (CDK4) was investigated at protein level in 104 ACC samples and tested by *in vitro* experiments in two ACC cell lines (NCI-H295R and MUC1). The most frequently overexpressed genes were *TOP2A* (100% of cases, median fold change = 16.5), *IGF2* (95%, fold change = 52.9), *CDK1* (80%, fold change = 6.7)*, CDK4* (62%, fold change = 2.6)*, PLK4* (60%, fold change = 2.8), and *PLK1* (52%, fold change = 2.3). CDK4 was chosen for functional validation, as it is actionable by approved CDK4/6-inhibitors (e.g., palbociclib). Nuclear immunostaining of CDK4 significantly correlated with mRNA expression (R = 0.52, *P* < 0.005). We exposed both NCI-H295R and MUC1 cell lines to palbociclib and found a concentration- and time-dependent reduction of cell viability, which was more pronounced in the NCI-H295R cells in line with higher CDK4 expression. Furthermore, we tested palbociclib in combination with insulin-like growth factor 1/insulin receptor inhibitor linsitinib showing an additive effect. In conclusion, we demonstrate that RNA profiling is useful to discover potential drug targets and that CDK4/6 inhibitors are promising candidates for treatment of selected patients with ACC.

## Introduction

Adrenocortical carcinoma (ACC) is a rare and aggressive tumor with generally poor but heterogeneous prognosis, which can be assessed by initial tumor stage and histopathological parameters like Ki67 proliferation index (1, 2). The presence of metastatic disease is associated with overall 5 year survival of <15% (1, 3, 4).

Current treatment options for ACC patients are scarce, with the only potential curative therapy being complete surgical resection (R0) (5). The adrenolytic drug mitotane is the only approved drug for treatment of ACC and can be administered as adjuvant therapy (6) or to patients with advanced disease (7). Cytotoxic chemotherapies such as etoposide-doxorubicin-cisplatin (EDP), streptozotocin and gemcitabine plus capecitabine represent further options but all show low response rates and frequent adverse effects (8–10). In addition, despite the proposal of some predictive markers of treatment response (9, 11), none of these have been applied clinically to predict responses in an individual patient.

Precision medicine represents an emerging approach in the field of cancer treatment. It involves molecular analyses to identify potentially targetable genes or pathways and then pre-select the most effective personalized therapy options. For ACC, biomarkers such as specific transcriptomic profiles, copy number alteration patterns and methylation in certain promoter regions have been identified by genome-wide studies to be associated with tumor aggressiveness and clinical outcome (12–15). In addition, previous molecular screenings have provided some promising insights into pharmacological targets, such as proteins involved in the cell cycle or tyrosine kinase receptors (2, 16, 17). The efficacy of some of these potential targets have previously been investigated in small clinical studies, but patients were not pre-selected and results were largely negative (18–23). Linsitinib, a dual inhibitor of the insulin-like growth factor 1 (IGF1R) and of the insulin receptor (IR), represents the only drug to have entered a phase III trial for ACC patients, but it also yielded disappointing results (24). However, it must be acknowledged that the molecular background of included patients in this trial was largely unknown.

Although our understanding of complex and heterogeneous ACC pathogenesis has improved through pan-genomic molecular studies over the last decade a targeted therapy is not yet available for patients with advanced disease. This concept has been recently well-summarized in a comprehensive review on genomic-guided clinical care in ACC (25). In a recent study, we demonstrated that a molecular screening performed by targeted next-generation sequencing on 107 available formalin-fixed paraffin-embedded (FFPE) tumor specimens was able to identify simultaneously prognostic markers and potential drug targets (2). The aim of the present study was to identify actionable genes and pathways at the mRNA level that might serve as a future personalized therapy in ACC. To this end, we performed targeted gene expression profiling on ACC tumor samples and functional *in vitro* studies.

## Materials and Methods

### Patient Cohort and Clinical Data

A total of 107 patients with histologically confirmed diagnosis of ACC and available DNA sequencing data from a previous publication were considered for this study (2). From these, 104 cases were included with available FFPE tumor specimens collected between 2002 and 2016. A total of 40 out of these 104 cases (33 primary tumors, 5 local recurrences, and 2 distant metastases) were also used for mRNA analysis (mRNA cohort, see below). Baseline clinical and histopathological characteristics, follow up information and details about pharmacological treatment (i.e., mitotane and/or cytotoxic chemotherapies) were collected through the ENSAT registry (https://registry.ensat.org//) and are summarized in Table 1. Furthermore, 9 normal adrenal glands (NAG) specimens and 11 adrenocortical adenoma (ACA) specimens were used as controls for immunohistochemistry analysis and 5 NAG as reference for gene expression analysis. The study protocol was approved by the local ethics committee (University Hospital of Wuerzburg, #88/11) and written informed consent was obtained from all subjects prior to study enrollment.

**Table 1:** Clinical and histopathological characteristics of patients with adrenocortical carcinomas in the entire cohort and in subgroup used for mRNA expression analysis (mRNA cohort).

	**Total cohort**	**mRNA cohort**
*n*	104	40
Sex (F/M)	59/45	25/15
Baseline		
Age—yrs (median, range) <50 years—*n (%)* ≥50 years—*n (%)*	49 (18–87) 54 (51.9) 50 (48.1)	46 (18–81) 24 (60.0) 16 (40.0)
Steroid secretion—*n* available Cortisol—*n (%)* Other single steroids (androgens, mineralocorticoids, or estrogens)—*n (%)* Mixed steroids—*n (%)* Inactive—*n (%)*	78 23 (22.1) 9 (8.7) 21 (20.2) 25 (24.0)	33 9 (22.5) 4 (10.0) 9 (22.5) 11 (27.5)
Tumor localization		
Primary tumor—*n (%)* Local recurrences—*n (%)* Metastases—*n (%)*	87 (83.7) 8 (7.7) 9 (8.6)	33 (82.5) 5 (12.5) 2 (5.0)
ENSAT tumor stage		
I-II—*n (%)* III—*n (%)* IV—*n (%)*	55 (52.9) 27 (26.0) 22 (21.1)	21 (52.5) 14 (35.0) 5 (12.5)
Resection status—*n* available R0—*n (%)* RX—*n (%)* R1—*n (%)* R2—*n (%)*	101 72 (69.2) 16 (15.4) 5 (4.8) 8 (7.7)	40 28 (70.0) 7 (17.5) 3 (7.5) 2 (5.0)
Ki67 proliferation index—median (range)	15 (2–90)	17.5 (3–90)
Follow-up		
Duration of follow up—months (median, range) Deaths	36 (1–280) 53 (51.0)	31 (4–280) 18 (45.0)
Therapeutic approaches		
Additional surgeries—*n (%)* Radiotherapy (tumor bed or metastases)—*n (%)*	38 (36.5) 32 (30.8)	18 (45.0) 15 (37.5)
Mitotane		
Adjuvant setting—*n (%)* Palliative setting—*n (%)*	38 (36.5) 38 (36.5)	16 (40.0) 14 (35.0)
Cytotoxic chemotherapies		
None—*n (%)* Platinum-based regimen—*n (%)* Streptozotocin—*n (%)* Gemcitabin plus capecitabin—*n (%)* Iodmetomidate—*n (%)*	41 (39.4) 53 (51.0) 43 (41.3) 37 (35.6) 4 (3.8)	17 (42.5) 18 (45.0) 19 (47.5) 14 (35.0) 4 (10.0)

*F, female; M, male; n, number of patients; R0, complete resection; R1, microscopic incomplete resection; R2, macroscopic incomplete resection; RX, uncertain resection; yrs, years*.

### RNA Isolation, Quality Testing, and Targeted mRNA Profiling

The tumor cell content of each FFPE slide was assessed by hematoxylin-eosin staining (median 90%, range 60–95%). RNA was isolated from tumors using miRNeasy FFPE kit (Qiagen, Hilden, Germany) according to manufacturer's instructions. One microgram of isolated RNA was reverse transcribed using the Quantitec Reverse Transcription Kit (Qiagen). RNA quality was determined by quantitative real-time PCR (RT-qPCR) for two housekeeping genes, *ACTB* (Hs9999903_m1) and *GAPDH* (Hs99999905_m1) (Applied Biosystems, Darmstadt, Germany), using the TaqMan Gene Expression Master Mix (Applied Biosystems), the CFX96 real-time thermocycler (Biorad, Hercules, CA, USA) and the Bio-Rad CFX Manager 2.0 software. Forty nanogram cDNA was used per reaction and run in duplicates. Cycling conditions were 95°C for 3 min, followed by 49 cycles of 95°C for 30 s, 60°C for 30 s, and 72°C for 30 s. A cycle threshold (CT) of ≤ 39 was required as quality test for targeted mRNA analysis. Accordingly, 40 samples qualified for further analysis (mRNA cohort) and were transcribed with the RT2 First Strand Kit (Qiagen) according to manufacturer's protocol. Expression of a panel of 84 drug targetable genes as well as five housekeeping genes (ACTB, B2M, GAPDH, HPRT1, RPLP0) and seven positive control genes was evaluated by the Human Cancer Drug Targets RT2 Profiler PCR Array (PAHS-507Z, Qiagen). The reaction was performed with the RT2 SYBR Green qPCR Mastermix (Qiagen). Cycling conditions were 95°C for 10 min followed by 40 cycles of 95°C for 15 s, 60°C for 1 min. Fold change (FC) was calculated with the 2^∧^(−ΔΔCT) formula normalized to five housekeeping genes and with a pool of five NAG from FFPE specimens as reference by the Qiagen GeneGlobe Data Analysis Center (https://www.qiagen.com/de/shop/genes-and-pathways/data-analysis-center-overview-page).

### Selection of Drug Target Candidate

We assessed the potential of the most frequently overexpressed genes as drug targetable events. First selection criterion was based on high frequency of gene overexpression in our ACC series (i.e., FC ≥2.0 in at least 50% of cases). According to this, we pre-selected a total of 6 candidates. The current stage of inhibitors targeting this gene candidates is listed in Table 2. Second selection criterion was the availability of specific inhibitors already approved by both U.S. Food and Drug Association (FDA) and/or European Medical Association (EMA) or at least in phase III clinical trials on solid tumors. Consequently, we choose cyclin-dependent kinase 4 (CDK4) as only ideal candidate for further investigation. We also checked for *CDK4* expression levels in previously published data sets from Affimetrix U133 Plus 2 chips that included 33 ACC as well as 22 adenomas and 10 NAG (GSE10297) (29) for further confirmation of our observation.

**Table 2:** Currently available inhibitors targeting the most frequently overexpressed genes reported in the present study.

**Gene**	**Available inhibitors (examples)**	**Current stage**
*IGF2*	IGFR/IR inhibitor (e.g., linsitinib)	Phase III trial in ACC patients (OSI-906)^a^
*TOP2A*	TOP2A inhibitors (e.g., aclarubicin)	Preclinical studies in ACC cells^b^
*CDK1*	Pan-CDK inhibitors (e.g., flavopiridol)	Phase I/II trials ongoing in solid tumors Preclinical studies in ACC cells^c^
*CDK4*	CDK4/6 inhibitors (e.g., palbociclib)	FDA and EMA approved for EGFR-negative breast cancer Phase II trials in liposarcoma Preclinical studies in ACC cells^d^
*PLK4*	PLK4 inhibitor (fumarate)	Phase I trials ongoing in solid tumors
*PLK1*	PLK1 inhibitor (e.g., TKM-080301)	Phase I/II trials ongoing in solid tumors

a Fassnacht et al. (24);

b Jain et al., (26);

c Nilubol et al., (27);

d Fiorentini et al. (28).

FDA, Food and Drug Association; EMA, European Medical Association.

*Sources: www.clinicaltrials.com, www.ema.europa.eu/en/medicines, www.fda.gov/drugs/informationondrugs/approveddrugs*.

### Immunohistochemistry

Immunohistochemistry (IHC) was performed on 2 μm thick sections of 104 ACC specimens, while 11 ACA and 6 NAG were additionally stained as controls. After deparaffinization, antigen retrieval was achieved by heating the slides for 13 min in the pressure cooker in 10 mM citric acid monohydrate buffer (pH 6.5). Unspecific binding sites were blocked with 20% human AB serum at room temperature (RT) for 1 h and slides were then incubated at RT for 1 h with specific antibodies against CDK4 (EPR4513-32-7, Abcam, Cambridge, United Kingdom, dilution 1:20) or N-Universal Negative control anti-rabbit (Dako, Golstrup, Denmark). Antibody binding was detected by means of the En-Vision System Labeled Polymer-HRP and developed for 10 min with DAB Substrate Kit (Vector Laboratories, Burlingame, CA, USA). Nuclei were counterstained with Mayer's hematoxylin. Other tissues, such as normal tonsil and thyroid carcinoma were included as positive controls.

Evaluation of stained slides was performed by two independent operators blinded to the results and clinical information (R.L. and S.St.). Intensity of nuclear staining and percentage of positive cells were rated as previously described, in order to calculate a semi-quantitative H-score (11). In case of discrepancies, slides were jointly assessed by both investigators and a final score was developed by consensus. Inter-observer agreement was determined by Pearson's correlation coefficient: 0.67 (95% CI = 0.55–0.76). The median CDK4 H-score was used for stratification of the patient cohort by CDK4 protein expression (*n* = 72 with CDK4 H-score ≤ 1 and *n* = 32 with CDK H-score >1).

### Adrenocortical Carcinoma Cell Lines and *in vitro* Experiments

CDK4 was selected to be investigated by *in vitro* experiments. Two ACC cell lines were used to test the efficacy of selected inhibitors targeting CDK4: the standard ACC cell line NCI-H295R and the newly established MUC1 cell line (30). Details about cell culture conditions as well as functional experiments are reported in Supplementary Table 1. In brief, to investigate genetic alterations and expression patterns of *CDK4* and its related genes, targeted next-generation sequencing was performed as previously published (2) and the Human Cancer Drug Targets RT2 Profiler PCR Array was obtained for both cell lines.

For CDK4 small interfering RNA (siRNA)-knock down, NCI-H295R or MUC1 cells were incubated with SMARTpool siRNA for CDK4. Moreover, cells were incubated with increasing drug concentrations of palbociclib (known CDK4/6 inhibitor, 0.5–16 μM) (31). Seventy two hours post-transfection with siRNA or upon completion of the drug treatment, cells were collected for RT-qPCR analysis or western blot (WB) analysis to demonstrate knock-down of CDK4 or examine changes in RNA and protein expression in the CDK4/6 pathway. The Hek293 and Hela cells were used as positive controls for *CDK4* and cyclin-dependent kinase inhibitor 2A (*CDKN2A*) mRNA expression, as previously published (32). Cell viability was assessed by the water-soluble tetrazolium (WST-1) reagent according to manufacturer's instructions (Roche Diagnostics Deutschland GmbH, Mannheim, Germany). In addition, cells were treated with increasing concentrations of dual IGF1R/IR inhibitor linsitinib (OSI-906, Selleckchem, 0.125–4 μM), which effectively reduces cell viability in NCI-H295R cells (33, 34). It is recognized IGF signaling may lead to activation of cyclin D by the phosphatidylinositol 3-kinase (PI3K)/ protein kinase B (AKT) pathway (**Figure 3C**). Inhibition of CDK4/6 and IGF1R achieved synergistic compromise of cell viability and proliferation while showing a correlation with reduced mammalian target of rapamycin complex 1 (mTORC1) activity (35, 36). For this reason, we decided to test the efficacy of CDK4/6 inhibitors in combination with linsitinib incubating both cell lines with a combination of palbociclib and linsitinib for 48, 96, 144, and 192 h. At least eight wells were used for each experimental condition and all experiments were conducted in triplicates.

### Western Blot (WB) Analysis

Details about WB analysis conditions are reported in Supplementary Table 1. In brief, after cell lysis, equal amount of proteins was loaded on a 4–20% gradient gel (BioRad) and separated by SDS-PAGE. The membrane was further probed with antibodies against CDK4 (EPR4513-32-7, abcam, dilution 1:1000) and CDKN2A (G175-405, BD Pharmingen, San Jose, CA, USA, dilution 1:500).

It is also known that CDK4, along with CDK6, activated by binding to D-type cyclins, is responsible for the G1-S phase transition in the cell cycle by phosphorylating and inhibiting the retinoblastoma (RB) protein and the related proteins p107/RBL1 and p130/RBL2 (36). Moreover, CDK4/6 inhibitors have been reported to reduce cell viability in ACC cell lines despite lack of pRb expression (28, 31) p130/RBL2 being already reported to be expressed in the NCI-H295R cell line (28). Thus, we investigated RB and p130/RBL2 protein expression in both cell lines. Signal detection was achieved by incubation with appropriate HRP-labeled secondary antibodies and Amersham ECL Prime reagent visualizing the protein-antibody complex by enhanced chemiluminescence.

### Statistical Analysis

A Fisher's exact or Chi-square test was used to investigate dichotomic variables, while a two-sided *t*-test or non-parametric Mann-Whitney test was used to compare two groups of continuous variables as appropriate. A non-parametric Kruskal-Wallis test followed by Bonferroni *post-hoc* test, was used for comparison among several groups for non-normal distributed variables. Correlations and 95% confidence intervals (95% CI) between different parameters were evaluated by linear regression analysis. Overall survival (OS) was defined as the time from the date of primary surgery to specific death or last follow-up, while progression-free survival (PFS) was defined as the time from the date of complete tumor resection to the first radiological evidence of disease relapse or disease-related death. Time to progression (TTP) during therapy was defined as the time from the date of first drug administration to the first radiological evidence of any kind of disease progression or relapse or death. Survival curves were obtained by Kaplan-Meier estimates and the differences between two or more curves were investigated by the log-rank (Mantel-Cox) test. A multivariate regression analysis including parameters with *p*-values below 0.1 at univariate analysis was performed by Cox proportional hazard regression model to identify the factors that might independently influence survival. Statistical analysis was performed with GraphPad Prism software 6.0 (GraphPad Software Inc., La Jolla, CA, USA) or SPSS software (IBM SPSS statistics, version 24). *P*-values below 0.05 were considered statistically significant.

## Results

### Overview of Gene Expression Profile (mRNA Cohort)

**Figure 1:**
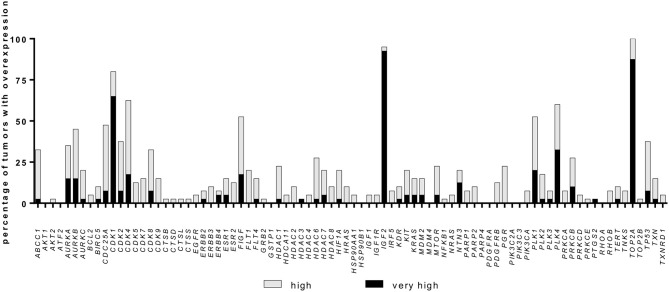
Gene overexpression in a cohort of 40 adrenocortical carcinoma samples. Percentage of samples with relative overexpression for 84 known cancer drug target genes (RT-qPCR profile, for details see Material and Methods). A fold change of ≥2.0 was defined as high expression whereas a fold change ≥5.0 was defined as very high expression.

Among the 84 investigated genes, 16 were relatively overexpressed with a FC ≥ 2.0 in at least 25% of samples, while 54 genes presented a FC <2 in at least 25% of samples. The percentage of samples with overexpression for each gene is shown in Figure 1. The six most frequently overexpressed genes were topoisomerase 2-alpha (*TOP2A*, 100% of cases, median FC 16.5, range: 2.0 to 126.7), insulin-like growth factor 2 (*IGF2*, 95% of cases, median FC 52.9, range: −58.6 to 532.9), *CDK1* (80% of cases, median FC 6.7, range: −1.3 to 27.4), *CDK4* (62% of cases, median FC 2.6, range: −1.5 to 15.0), polo like kinase (*PLK*) *4* (60% of cases, median FC 2.8, range: −1.6 to 36.0), and *PLK1* (52% of cases, median FC 2.3, range: −2.7 to 33.9).

Additionally, some gene families were found to be upregulated in more than 50% of samples: Aurora Kinase (AURK, 62.5% of cases with at least one member overexpressed), CDK (95% of cases) and PLK (75% of cases, Figure 1). Notably, while *IGF2* was observed to be strongly overexpressed, both *IGF1* and *IGF1R* were downregulated in most cases (median FC −2.9, range: −67.8 to 3.0, and median FC −1.7, range: −25.5 to 3.0, respectively). *CDK4* expression levels were not associated with *IGF2, IGF1* nor *IGF1R* levels, but 37.5% of cases presented high CDK4 and normal/high IGF1R expression levels (Supplementary Figures 1A,B).

Furthermore, we observed a significant positive correlation between Ki67 proliferation index and mRNA expression of *AURKB*, cell division cycle 25A (*CDC25A*)*, CDK1, CDK2*, fms-related tyrosine kinase 1 (*FLT1*), histone deacetylase 2 (*HDAC2*)*, MTOR*, poly (ADP-ribose) polymerase 1 (*PARP1*), platelet derived growth factor receptor beta (*PDGFRB*) and *TOP2A* (data not shown).

### CDK4 as Promising Drug Target for ACC

**Figure 2:**
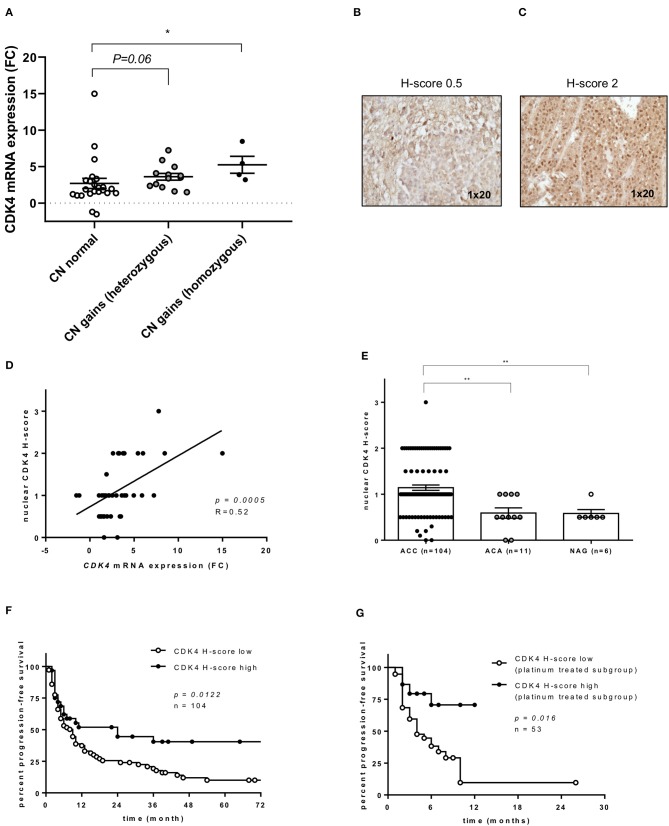
*CDK4* gene expression and CDK4 nuclear protein expression in adrenocortical carcinoma (ACC). **(A)** Relationship between *CDK4* gene expression and copy number (CN) status at DNA level (*n* = 40) (2): *CDK4* mRNA fold changes (FC) in ACC with normal CN status (*n* = 23) or *CDK4* CN gain heterozygous (*n* = 13) or homozygous (*n* = 4) as previously published. Bars represent median and interquartile range. *p < 0.05. Statistical analysis by Kruskal-Wallis test followed by Bonferroni *post-hoc* test. **(B,C)** Examples of CDK4 immunostaining in two ACC samples, one with negative nuclear staining (H-score = 0) and one with intermediate positive staining (H-score = 2). Magnification 1 × 20. **(D)** Relationship between *CDK4* mRNA FC and CDK4 nuclear protein expression evaluated by immunohistochemistry (as H-score) (*n* = 40). The regression line is shown. Statistical analysis by Pearson *r* correlation test. **(E)** CDK4 nuclear protein expression evaluated by immunohistochemistry (as H-score) in normal adrenal glands (NAG, *n* = 6), adrenocortical adenomas (ACA, *n* = 11) and ACC samples (*n* = 104) (*p* < 0.0002 per trend). Bars represent median and interquartile range. **p < 0.01. Statistical analysis by Kruskal-Wallis test. **(F)** Progression-free survival curves comparing low CDK4 expression defined as H-score ≤ 1 (*n* = 72) and high expression defined as H-score > 1 (*n* = 32). Statistical analysis by log-rank test. **(G)** Time to progression in platinum-treated ACC cohort (*n* = 53) comparing low expression (*n* = 38) and high expression (*n* = 15) as defined above. Statistical analysis by log-rank test.

According to our selection criteria, CDK4 was chosen as the most promising targetable drug candidate. Alterations in cell cycle regulation are frequently observed in ACC development and considered to be important ACC drivers. In particular, cell cycle alterations related to the p53/Rb1 pathway constitute an attractive target for cancer therapy (37). Of note, *CDK4* copy number (CN) gains, as well as losses of its regulator, *CDKN2A*, are frequently reported in ACC (14–16). In our entire ACC cohort (*n* = 104), previously analyzed by targeted next generation sequencing, CN gains for *CDK4* were observed in 43% of cases (2). Comparing *CDK4* CN status and mRNA expression, ACC with normal CN status (*n* = 24) showed significantly lower mRNA expression than ACC with *CDK4* CN gains (*n* = 16) (*p* = 0.0085 by Kruskal-Wallis test, Figure 2A). In addition, looking at available transcriptome data sets (29), a significantly higher *CDK4* expression can be likewise observed in ACC vs. both adenomas and NAG (Supplementary Figure 2).

### CDK4 Protein Expression: Relationship With Gene Expression and Clinical Outcome

CDK4 expression was evaluated by immunostaining in our entire ACC series (*n* = 104). Two representative examples for CDK4 immunostaining in ACC samples are shown in Figures 2B,C, while the cohort stratification by low and high CDK4 nuclear H-score is reported in the Supplementary Table 2. Considering separately the mRNA cohort (*n* = 40), a significant positive correlation between *CDK4* mRNA and nuclear protein expression was observed (*p* = 0.0005, R = 0.52, Figure 2D). However, only a positive trend was detected between CN status and CDK4 protein expression in the entire cohort (*n* = 104, *p* = 0.2285, data not shown). Overall, we observed a significantly higher nuclear CDK4 expression (H-score) in ACC (*n* = 104) than in both ACA (*n* = 11, *p* < 0.01) and NAG (*n* = 6, *p* < 0.01), while no difference was observed between ACA and NAG (Figure 2E).

Considering the clinical outcome (*n* = 104 ACC), OS showed no relationship with CDK4 protein expression (*p* = 0.2013, data not shown), while PFS was slightly longer in patients with high CDK4 nuclear expression compared to patients with low expression (median=24 vs. 9 months, *p* = 0.0122, HR = 0.56, 95% CI = 0.36–0.88, Figure 2F). As expected, PFS was also significantly associated with ENSAT tumor stage (stage 1–2 vs. 3–4, *p* = 0.001, HR = 2.25, 95% CI = 1.39–3.64), Ki67 proliferation index (cut-off 15%, *p* < 0.001, HR = 3.53, 95% CI = 2.11–5.89), and resection status (R0-X vs. R1–2, *p* < 0.001, HR = 6.49, 95% CI 2.5–16.8). Of note, including these parameters in a multivariate analysis, the relationship between CDK4 expression and PFS remained statistically significant (*p* = 0.044, HR = 0.45, 95% CI = 0.20–0.98). Further investigation showed that high CDK4 protein expression was significantly associated with longer TTP during treatment with platinum-based chemotherapy (*n* = 53, median 6 vs. 4 months, *p* = 0.0156, HR = 3.1, 95% CI = 1.4–6.7) (Figure 2G). No other significant relationship was observed between CDK4 immunostaining and response to therapy.

### Expression of CDK4-Related Genes and Proteins in ACC Cell Lines

**Figure 3:**
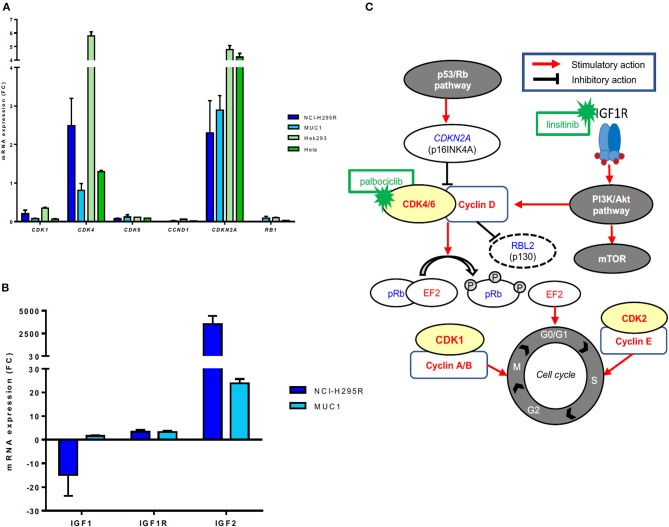
Gene expression of CDK4-related factors in different adrenocortical carcinoma (ACC) cell lines. **(A)** mRNA expression levels of *CDK1, CDK4, CDK6, CCND1, CDK2NA*, and *RB1* in ACC cell lines NCI-H295R and MUC1 cells (triplicates by RT-qPCR). Hek293 and Hela cells were used as positive controls and β-actin was used as housekeeping gene as internal standard. **(B)** Schematic representation of the cyclin-dependent kinases/cyclin complexes, their regulation by mitogenic signals and their role in regulating the cell cycle progression as well as corresponding available inhibitors. Activating factors are represented in red, suppressor factors in blue. **(C)** mRNA expression levels of *IGF1, IGF1R*, and *IGF2* in ACC cell lines NCI-H295R and MUC1 cells (triplicates by RT-qPCR). β-actin was used as housekeeping gene as internal standard.

No CN variations in the genes *CDK4* and *CDKN2A* were observed in either NCI-H295R or MUC1 cells. *RB* gene losses were found in NCI-H295R cells, but not in MUC1 cells. A specific mRNA profile of CDK4-related genes was obtained and validated on protein level (Figures 3A,B). In particular, *CDK4* mRNA expression was relatively high in NCI-H295R (2.50 ± 1.01) and moderate in MUC1 cells (0.81 ± 0.25), these findings being reinforced by WB analysis in comparison to other CDK4 expressing cell lines as positive controls (Supplementary Figure 3). A rather high *CDKN2A* mRNA expression was detected in both NCI-H295R (2.30 ± 1.19) and in MUC1 (2.89 ± 0.53) cell lines, while CDKN2A/p16^INK4A^ protein expression was clearly lower in NCI-H295R cells than MUC1 cells. No mRNA expression of *RB1* was observed in NCI-H295R and expression was low in MUC1 cells (0.09 ± 0.06), while no RB1 protein expression was shown in both cell lines. On the other side, p130/RBL2 was consistently expressed in both ACC cell lines. *CDK1, CDK6*, and *CCND1* mRNA were expressed at low levels in both ACC cell lines (all <0.4).

Additionally, RT profiling showed a very strong *IGF2* overexpression (FC > 2,000) and a strong *IGF1R* overexpression (FC = 4.1) in NCI-H295R cells associated with a downregulation of *IGF1* (FC = −32). MUC1 cells presented *IGF2* (FC >20.0) and *IGF1R* (FC = 2.8) overexpression and regular expression of *IGF1* (FC = 1.2) (Figure 3C).

### CDK4 Inhibition by siRNA in ACC Cell Lines

NCI-H295R cells and MUC1 cells were successfully transfected with *CDK4* siRNA. 72 h after transfection, *CDK4* was significantly reduced in comparison to mock transfected control cells in both cell lines at mRNA (60% reduction, *p* = 0.0175, and 85% reduction, *p* = 0.0022, for NCI-H295R cells and MUC1 cells, respectively, Supplementary Figure 4A) and protein level (80% reduction, *p* = 0.0039, and 95% reduction, *p* = 0.0004, for NCI-H295R cells and MUC1 cells, respectively, Supplementary Figure 4B). WST1 assays were performed to examine the impact of *CDK4* downregulation on cell viability. Whereas, *CDK4* knockdown in NCI-H295R showed a significant decrease of cell viability when compared to the control (88.6 ± 9.3 vs. 100.0 ± 8.6, *p* < 0.0001) (Supplementary Figure 4C), no clear effect was detected in MUC1 cells (97.0 ± 7.6 vs. 100.0 ± 6.9, *p* = 0.175) (Supplementary Figure 4C).

### Treatment With CDK4/6 Inhibitor Palbociclib in ACC Cell Lines

**Figure 4:**
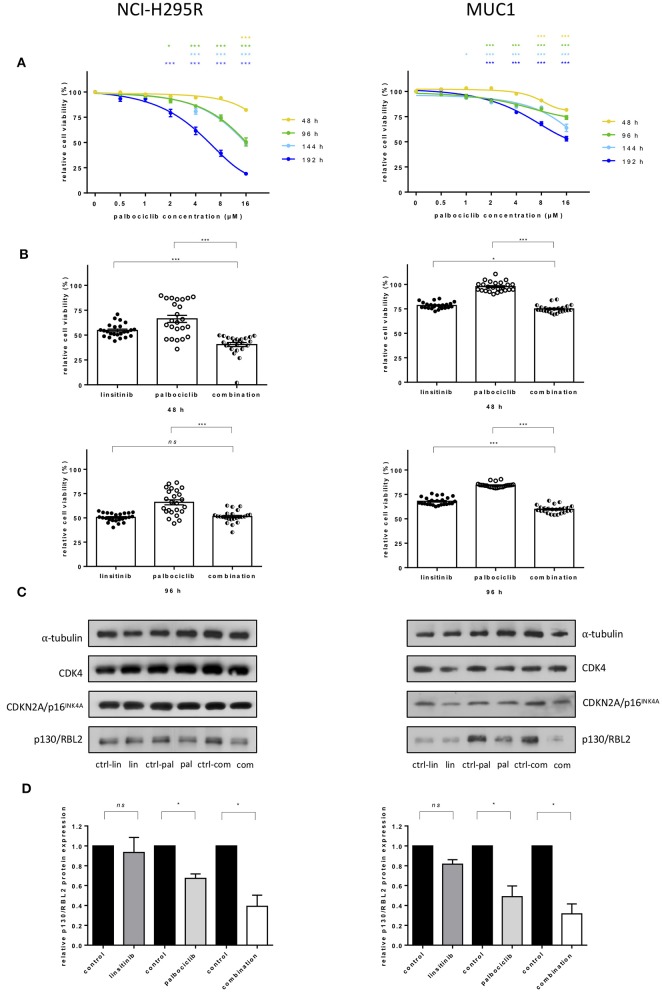
Effects of treatment with CDK4/6 inhibitor palbociclib alone and in combination with linsitinib in adrenocortical cell lines: NCI-H295R (left) and MUC1 (right). **(A)** Interpolation of cell viability measured by WST1 test in 48 h intervals after administration of ascending concentrations of palbociclib. The mean of the absorbance measured for cells treated with DMSO was defined as 100% for each experiment. The ratio of measured absorbance to the mean absorbance forms the final data. For both cell lines three independent experiments were conducted using octuplet samples. *p <0.05, ***p <0.001 for comparison with the control sample. Statistical analysis by two-way ANOVA. **(B)** Cell viability under additional administration of 0.25 μM linsitinib and 4 μM palbociclib in NCI-H295R and MUC1 cells after 48 and 96 h. ns = p not significant, *p <0.05, ***p <0.001. Statistical analysis by one-way ANOVA. **(C)** Western blot analyses show little effect after drug treatment for 96 h on protein expression of CDK4 and CDKN2A/p16^INK4A^ but decreasing levels of p130/RBL2 after palbociclib treatment as well as after treatment with the combination of palbociclib and linsitinib in both cell lines. The blot is representative for three independent experiments. ctrl-lin, correspondent control to linsitinib treatment; lin, linsitinib; ctrl-pal, correspondent control to palbociclib treatment; pal, palbociclib treatment; ctrl-com, correspondent control to combination treatment; com, combination of palbociclib and linsitinib. **(D)** Quantitative western blot analysis of p130/RBL2 in linsitinib, palbociclib and combination treated cells. α-tubulin was used as the internal standard. Each corresponding control was defined as 1.0, each bar of the histograms represents the relative ratio of p130/RBL2 to α-tubulin signal after normalization to the control. Each experiment was conducted in triplicates. ns = p not significant, *p <0.05. Statistical analysis by unpaired *t*-test with Welch's correction.

To assess the efficacy of palbociclib *in vitro*, the drug was administered in increasing concentrations to NCI-H295R and MUC1 cell lines. A time and dose dependent reduction of cell viability was observed. Specifically, in NCI-H295R cells, treatment with 2 μM palbociclib reduced cell viability after longer time intervals (96 and 192 h), while administration of higher concentrations (i.e., 16 μM) was already able to achieve a decrease in cell viability at 48 h compared to vehicle treated cells (82.3 ± 5.2 vs. 50.5 ± 19.2 vs. 48.9 ± 4.3 vs. 19.1 ± 4.6 at 48, 96, 144, 196 h, respectively, all *p* < 0.001) (Figure 4A). Similar results were obtained in MUC1 cells, where a reduction of cell viability was observed with both 8 and 16 μM palbociclib at all time points (81.7 ± 3.0 vs. 74.1 ± 8.7 vs. 63.8 ± 17.7 vs. 53.1 ± 10.9 for 16 μM palbociclib at 48, 96, 144, 196 h, respectively, all *p* < 0.001) (Figure 4A). However, comparing the general effect of palbociclib in the two ACC cell lines, a stronger decrease in cell viability was observed in NCI-H295R which was obvious at higher drug concentrations or longer treatment times (Supplementary Figure 5A).

### Combined Treatment With Palbociclib and Linsitinib in ACC Cell Lines

Linsitinib alone significantly reduced cell viability in a dose- and time-dependent manner in both cell lines (Supplementary Figure 5B). To investigate a potential combination of both palbociclib and linsitinib, 0.25 μM linsitinib and 4 μM of palbociclib were administered to both NCI-H295R and MUC1 cells and cell viability was measured at 48 and 96 h. Viability in NCI-H295R cells was significantly lower in cells treated for 48 h with a combination of both drugs than in single drug treatment (40.5 ± 9.7 vs. 54.7 ± 6.5 and 66.3 ± 15.6 for combination vs. linsitinib and palbociclib, respectively, both *p* <0.001) (Figure 4B). At 96 h a significant reduction of cell viability remained only in comparison to palbociclib treatment (51.4 ± 5.8 vs. 50.5 ± 4.2 and 66.0 ± 12.1 for combination vs. linsitinib and palbociclib treatment, respectively, ns and *p* <0.001). In MUC1 cells, treatment with the combination showed a significant reduction in cell viability in comparison to linsitinib (74.9 ± 3.6 vs. 78.5 ± 3.1, *p* <0.05) and palbociclib (97.7 ± 4.8, *p* <0.001) alone at 48 h. Significance of the effect of the combination in respect to linsitinib alone increased (59.8 ± 3.8 vs. 68.0 ± 3.8, *p* <0.001) at 96 h while comparison with palbociclib remained unchanged (84.3 ± 2.3, *p* <0.001).

### Effects of Palbociclib and Linsitinib on CDK4 Pathway

To better understand the effect of the drugs on the CDK4 pathway, we investigated protein levels of CDK4, CDKN2A/p16^INK4A^, and p130/RBL2 in cells after treatment with palbociclib and linsitinib alone or in combination (Figures 4C,D). No difference was shown in terms of CDK4 and CDKN2A/p16^INK4A^. Contrarily, p130/RBL2 was significantly decreased after palbociclib and combined treatment in both cell lines NCI-H295R and MUC1, respectively (Figure 4D).

## Discussion

Despite intensive efforts and promising preliminary findings, no effective targeted treatment is available for patients with advanced ACC. In this study, we aimed to identify new potential targeted therapies that might be used in the clinical setting in the near future. To this end, we performed targeted gene expression profiling on 40 standard FFPE tumor specimens focusing on known anti-cancer drug targets. We identified promising actionable gene families, such as CDK, PLK, and AURK families. Considering the fact that (1) the *CDK4* gene was overexpressed in 62% of ACC samples, (2) CDK4/6 inhibitors such as palbociclib are already approved for breast cancers and under clinical trials for other solid tumors, (3) *CDK4* expression correlated with the presence of CN gains at DNA level (2), and (4) palbociclib has been previously tested in ACC cell lines with promising results (28, 31), we decided to focus on CDK4 as our best potential drug target for ACC. We also evaluated CDK4 protein expression by immunohistochemistry in a large series of 104 ACC samples and observed a strong correlation with mRNA levels. In addition, CDK4 nuclear expression was associated to longer PFS, even if not to longer OS. A similar association regarding CDK4/6 specific activity was reported in high-risk endometrial cancer (38). Additionally, we observed a correlation between high CDK4 immunoreactivity and longer TTP during treatment with platinum-based chemotherapy. CDK4 has previously been reported to be one of the targets of cisplatin. High CDK4 expression could therefore be involved in better response to therapy with platinum compounds (39) and thus explain at least in part the longer PFS observed in the entire cohort. More commonly, however, CDK4 is related to unfavorable prognosis in several solid tumors (40). A larger cohort is definitely needed for a sound statement on the prognostic role of CDK4 in ACC patients. Altogether, even if high CDK4 levels might be associated with favorable clinical outcome, we are convinced that there is a strong rationale for using palbociclib in this group of ACC patients. Indeed, about 50% of them will have a recurrence within the first 2 years after primary surgery and a good proportion of them will need effective palliative pharmacological treatment.

We then performed functional *in vitro* studies in order to test the effects of CDK4 inhibition on cell viability in two different ACC cell lines (NCI-H295R and MUC1) (30). CDK4/6 inhibitors (i.e., abemaciclib, palbociclib, and ribociclib) were recently approved for hormone receptor positive, human epidermal growth factor receptor 2 (EGFR)-negative, advanced breast cancer in combination with hormone therapy (41, 42). Currently, they are also under investigation in phase I and II clinical trials on other solid tumors (43, 44) and in patients with amplification or overexpression of *CDK4* at tumor level (45) (NCT03242382). Two recent studies on ACC cell lines already showed the effect of CDK4/6 inhibitors on cell viability. While Fiorentini et al. described the effect of palbociclib in dependence with CDK4 and CDK6 mRNA overexpression in NCI-H295R and SW13 cell lines (28), Hadjadj et al. investigated palbociclib mainly under consideration of CDK6 expression (31). In our study, we could confirm a concentration- and time-dependent cell viability reduction through palbociclib in NCI-H295R cells and for the first time also in the novel ACC cell line MUC1. Of note, we observed that this effect was more evident in NCI-H295R than in MUC1 cell line. This effect might be at least in part due to the higher CDK4 expression observed in NCI-H295R cells in contrast to MUC1 cells.

In terms of mechanisms, consistent with previous studies reporting the deletion of the *RB* gene and lack of RB1 in a subset of ACC tumors (14, 46), we found no RB1 protein expression in neither NCI-H295R nor MUC1 cells, similarly to RB negative palbociclib sensitive hepatoma cells. Therefore, we focused our attention on p130/RBL2, a member of the RB-like family, previously reported to be expressed in NCI-H295R cells (28, 31, 47). Indeed, we could show notable p130/RBL2 expression in NCI-H295R as well as in MUC1 cells and also observed a relative decrease of the p130/RBL2 protein in treated cells indicating that CDK4 inhibition by palbociclib could actually lead to an activation of p130/RBL2, which is involved in cell cycle arrest and senescence (48).

However, present and previous studies on palbociclib in ACC cell lines (31) utilized rather high drug concentrations (i.e., 1–5 μM) which would be difficult to maintain in patients, implying that further investigations in animal models might be needed to confirm the anticancer effects of palbociclib for ACC.

IGF signaling is known to be activated in about 80–90% of cases (49, 50) and the IGFR/IR inhibitor linsitinib has been demonstrated to be a promising targeted therapy for ACC by *in vitro* experiments (33). However, these encouraging findings have not been confirmed in clinical trials, although a small percentage of patients showed a partial response to treatment (24). In the present series, we confirmed *IGF2* overexpression in 95% of cases, whereas *IGF1R* was down-regulated in 45% of cases, similarly as previously described (34). Of note, however, 37.5% of cases presented high CDK4 and normal or high IGF1R mRNA levels. On the other hand, an *IGF1R* overexpression is found in NCI-H295R cells in our and previous studies (33, 34). Thus, with IGF1R being the main target of linsitinib, its low/absent expression in ACC might be one reason for the disappointing response rate in ACC patients (24) and pre-screening for *IGF1R* overexpression might then be useful to pre-select patients that may benefit from this treatment. Another reason for linsitinib failure might be represented by resistance mechanisms related to CDK4/6 activation. In fact, it is recognized that IGF signaling activation can lead to upregulation of cyclin D1 and activation of CDK4/6 (51). On this connection, we tested a combination of palbociclib and linsitinib in ACC cell lines to examine the effect of targeting two pathways simultaneously. Concurrent inhibition of IGF1R and CDK4/6 has shown a greater decrease in cell viability than achieved by a single drug in both NCI-H295R and MUC1 cells, as reported in different tumor cell lines (35, 52, 53). Currently, a phase Ib clinical trial with an IGF1/IGF2 antibody and CDK4/6 inhibitor is ongoing in different solid tumors (NCT03099174). To date, mitotane or the combination of mitotane with the cytotoxic regimen of etoposide, doxorubicin and cisplatin (EDP-M) is recommended as first-line treatment of advanced ACC (8). Mitotane is generally considered as a toxic drug with a narrow therapeutic range, adverse events often limiting the dosage mostly include gastrointestinal disorders and neurological effects (5). EDP-M is currently the most validated option for advanced ACC, however, response rates remain low. Common adverse events are hematological, gastrointestinal and neurological effects (5, 8). Both palbociclib and linsitinib were reported to be mostly well-tolerated with the most common adverse events being hematologic toxicity under palbociclib (54) and fatigue, nausea and hyperglycemia under linsitinib (24). Thus, even a combination of Palbociclib and linsitinib remains theoretically competitive compared to EDP-M.

In conclusion, our easy-to-apply screening approach allowed us to identify promising target genes that might serve for personalized management of patients with advanced ACC. In particular, it seems to be feasible in the near future to pre-select ACC patients that might potentially benefit from a treatment with CDK4/6 inhibitors, consistent with the presence of CDK4 CN gains (at DNA level, 43% of cases) and/or CDK4 overexpression (at mRNA or protein level) in the tumor, while linsitinib might be an interesting combination partner in patients with both *IGF2* and *IGF1R* overexpression. Our study is the preclinical basis for a clinical trial investigating CDK4/6 inhibitors in ACC, a disease in which personalized therapeutic approaches are urgently needed.

## Data Availability Statement

The data can be found in NCBI's Bioproject repository with accession number - PRJNA596100.

## Ethics Statement

The studies involving human participants were reviewed and approved by University Hospital of Wuerzburg (Germany). The patients/participants provided their written informed consent to participate in this study.

## Author Contributions

RL participated to the design of the study, performed the experiments, collected the clinical data, performed part of the statistical analysis, and wrote the manuscript. IW contributed to the *in vitro* experiments, to the interpretation of the results, and to write the manuscript. JL performed the genetic analysis on ACC cell lines. SK provided the tissue samples from pathology and contributed to the immunohistochemistry analysis. BA contributed to the collection of the clinical data, to the interpretation of the results, and to write the manuscript. SSt contributed to the immunohistochemistry analysis. CH provided the MUC1 cell line and contributed to the interpretation of the results. SR supervised the genetic analysis. AR supervised the immunohistochemistry analysis. MK and MF contributed to the interpretation of the results and to write the manuscript. SSb supervised the *in vitro* experiments, contributed to the interpretation of the results, and to write the manuscript. CR designed the study protocol, supervised the experiments, and contributed to the interpretation of the results and the writing of the manuscript.

### Conflict of Interest

The authors declare that the research was conducted in the absence of any commercial or financial relationships that could be construed as a potential conflict of interest.
