# Supplementary figures and images for: Targeted Gene Expression Profile Reveals CDK4 as Therapeutic Target for Selected Patients With Adrenocortical Carcinoma
Source: Front Endocrinol (Lausanne). 2020 Apr 16;11:219. doi: 10.3389/fendo.2020.00219 (PMC7176906; doi:10.3389/fendo.2020.00219)

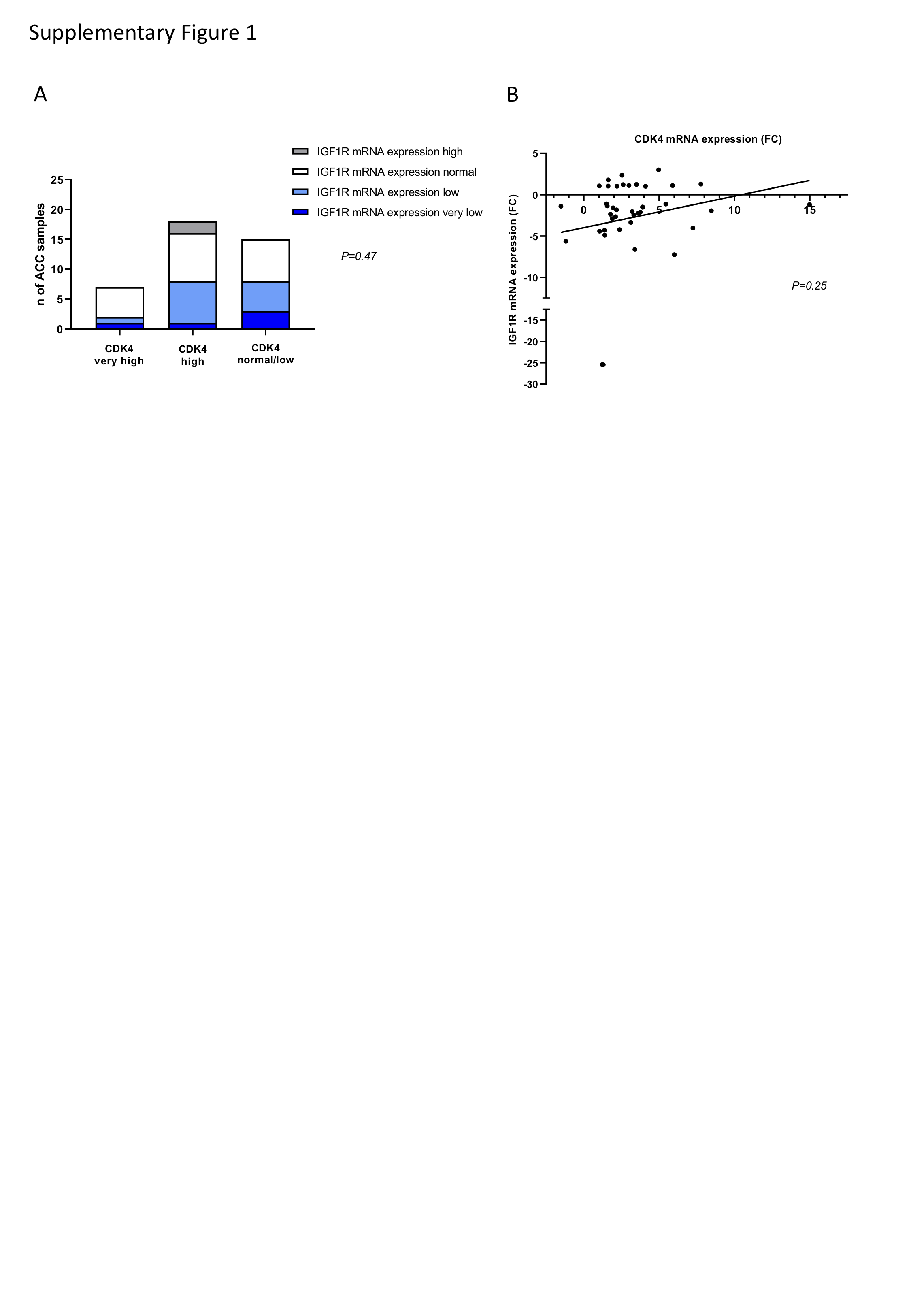

Supplement: Supplementary file 1 [file Image_1.TIFF]

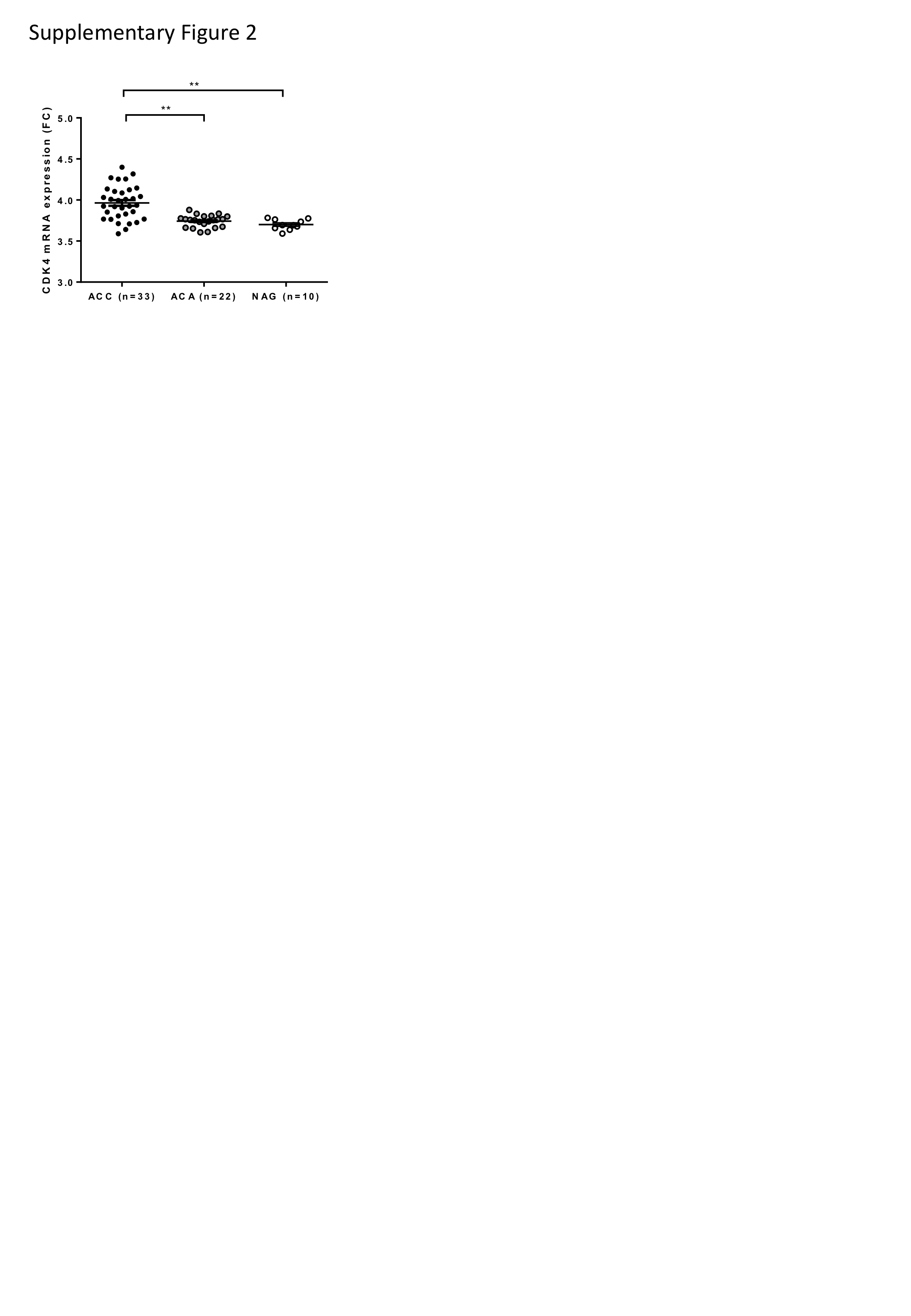

Supplement: Supplementary file 2 [file Image_2.TIFF]

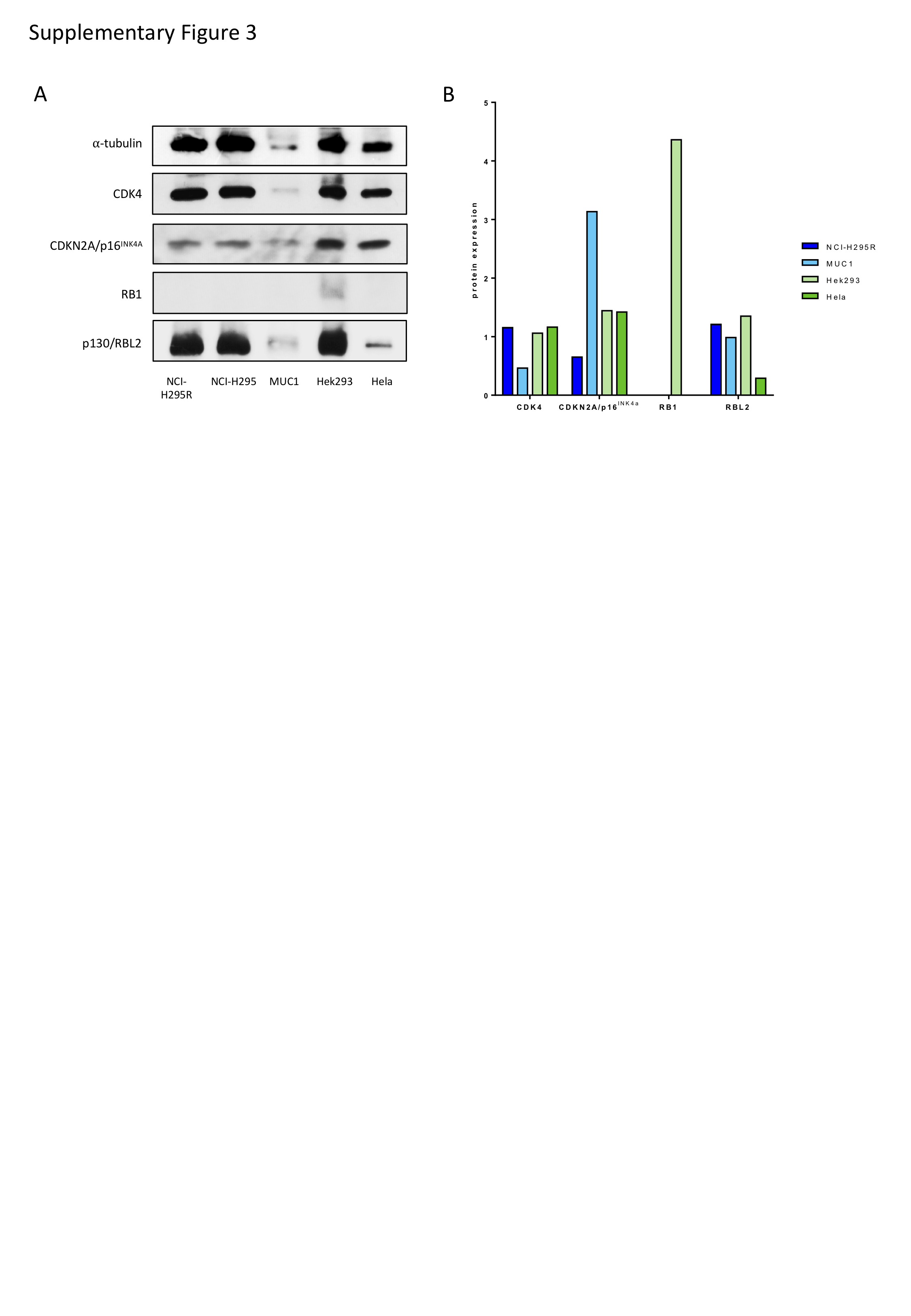

Supplement: Supplementary file 3 [file Image_3.TIFF]

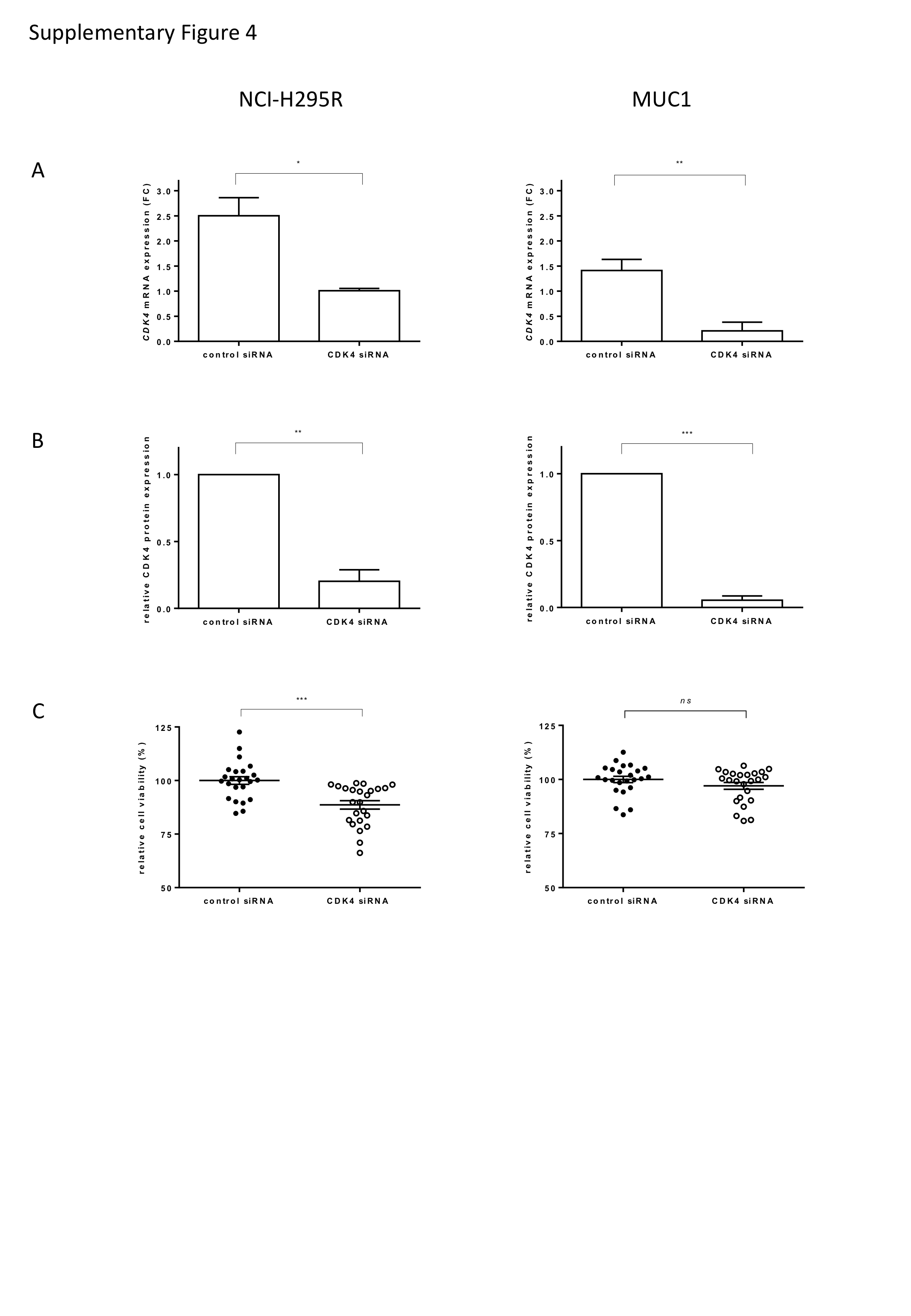

Supplement: Supplementary file 4 [file Image_4.TIFF]

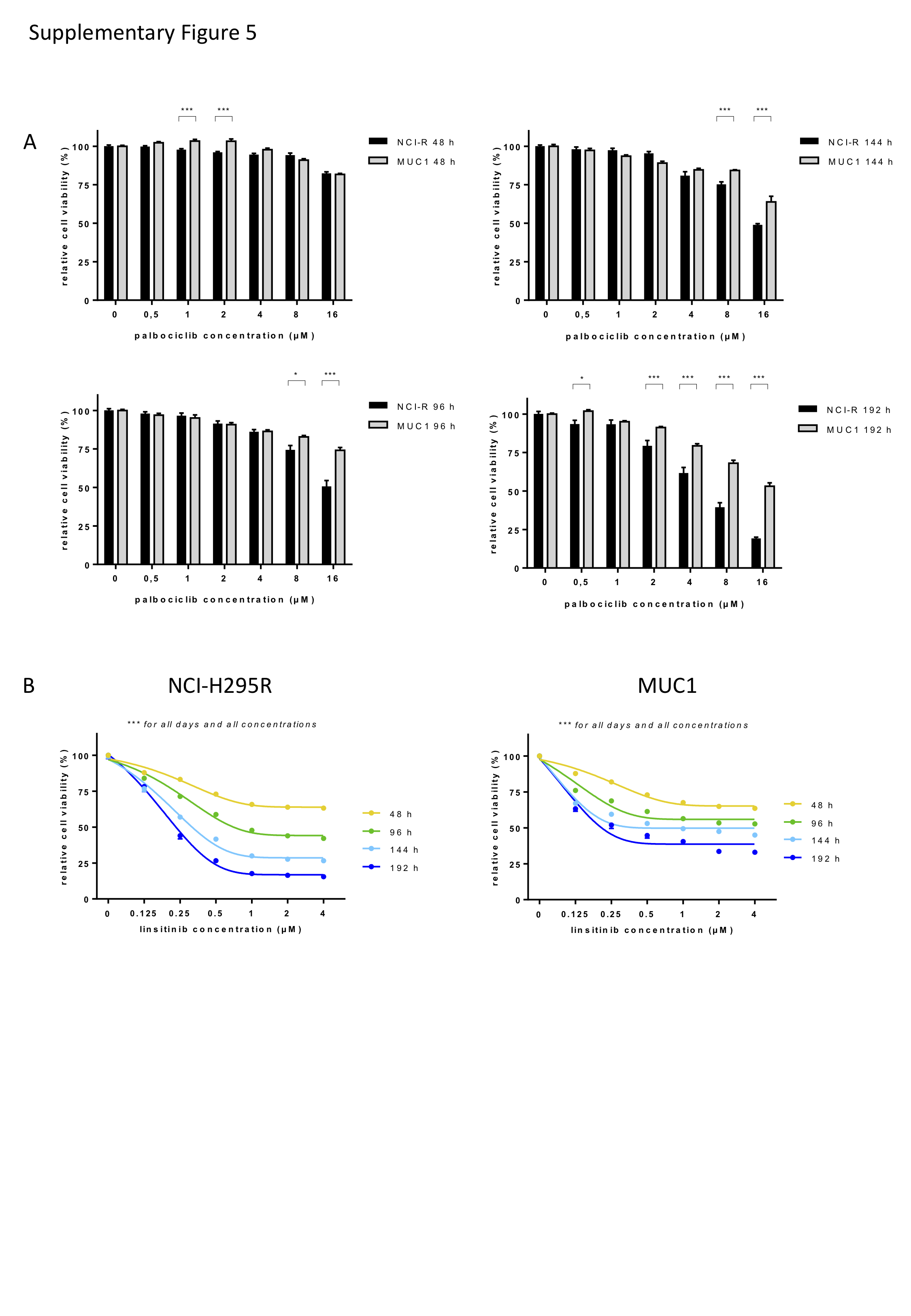

Supplement: Supplementary file 5 [file Image_5.TIFF]
