# Supplementary material for: Targeted Gene Expression Profile Reveals CDK4 as Therapeutic Target for Selected Patients With Adrenocortical Carcinoma
Source: Front Endocrinol (Lausanne). 2020 Apr 16;11:219. doi: 10.3389/fendo.2020.00219 (PMC7176906; doi:10.3389/fendo.2020.00219)
Supplement: Supplementary file 6 [file Data_Sheet_1.docx]

Supplementary Material

# Supplementary Tables

**Supplementary Table 1. Adrenocortical carcinoma cell lines and *in vitro* experiments**

| **Adrenocortical carcinoma cell lines** |
| --- |
| NCI-H295R cells were maintained in DMEM medium supplemented with 2.5% Nu-Serum and 5ml Insulin-Transferrin-Selenium, while MUC1 cells were cultured in Advanced DMEM medium supplemented with 10% FCS and penicillin/streptomycin. Both cell lines were cultivated in flasks in a humid atmosphere at 37°C and 5% CO_2_ and were used between passages 9 and 15. Short-tandem repeat profiling confirmation was performed on both cell lines and mycoplasma contamination PCR tests have been performed regularly to maintain mycoplasma free. |
| **DNA isolation and NGS** |
| DNA was isolated from both cell lines by DNeasy Blood & Tissue Kit (Qiagen) and targeted next-generation sequencing was performed as previously published (Lippert et al., 2018). Moreover, RNA was extracted using the Maxwell® RSC simplyRNA Tissue Kit (Promega, Madison, WI, USA) and the Human Cancer Drug Targets RT2 Profiler PCR Array was obtained for both cell lines. Three NAG were used for reference. |
| **CDK4 small interfering RNA (siRNA)-knock down** |
| 2.5*10^4 NCI-H295R cells or 2*10^4 MUC1 cells were seeded in a 96-well plate one day prior to transfection and incubated with 1µM SMARTpool siRNA for CDK4 (L-003238-00-0005, Dharmacon, Cambridge, United Kingdom) according to manufacturer’s recommendations. Non-coding siRNA pool (Dharmacon) was used as negative control. |
| **Treatment with palbociclib (CDK4/6 inhibitor)** |
| Cells were plated for drug treatment with palbociclib (Selleckchem, Houston, TX, USA). Cells were incubated with increasing drug concentrations of palbociclib (0.5-16μM) (Hadjadj et al., 2017). Generally, drug-containing medium was replaced every other day and the diluent DMSO was used as control treatment. 72h post-transfection with siRNA or upon completion of the drug treatment, cells were collected for RT-qPCR analysis or western blot (WB) analysis to demonstrate knock-down of CDK4 or examine changes in RNA and protein expression in the CDK4/6 pathway. |
| **Gene expression analysis** |
| Gene expression of *CDK4* (Hs00364847_m1) or additional *CDK4*-related genes including *CDK1* (Hs00938777_m1), *CDK6* (Hs01026371_m1), *CDKN2A* (Hs00923894_m1), *CCND1* (Hs00765553_m1) and *RB1* (Hs01078066_m1) were analyzed by RT-qPCR using the TaqMan Gene Expression Master Mix (Applied Biosystems), the CFX96 real-time thermocycler (Biorad) and the Bio-Rad CFX Manager 2.0 software. The Hek293 and Hela cells were used as positive controls for *CDK4* and cyclin-dependent kinase Inhibitor 2A (*CDKN2A*) mRNA expression, as previously published (Sun et al., 2013). |
| **Cell viability test** |
| Cell viability was assessed by the water soluble tetrazolium (WST-1) reagent according to manufacturer’s instructions (Roche Diagnostics Deutschland GmbH, Mannheim, Germany). After a 2h incubation with WST1 reagent, absorbance was measured at 450nm by a microplate reader (1420 VICTOR3, PerkinElmer Inc, Waltham, MA, USA). |
| **Treatment with linsitinib (dual IGF1R/IR kinase inhibitor)** |
| Cells were treated with increasing concentrations of dual IGF1R/IR kinase inhibitor linsitinib (OSI-906, Selleckchem, 0.125-4µM). The efficacy of CDK4/6 inhibitors in combination with linsitinib was tested incubating both cell lines with a combination of palbociclib and linsitinib for 48h, 96h, 144h and 192h. |
| **Western Blot Analysis** |
| Cells were lysed by incubation with RIPA buffer (Sigma-Aldrich, St. Louis, MO, USA). Equal amount of proteins was loaded on a 4-20% gradient gel (BioRad) and separated by SDS-PAGE at 80V for 20 min and 150V for 45 min. Proteins were transferred on a nitrocellulose membrane by tank-blot. After blocking with 5% skimmed milk in TBS-Tween at RT for 1h, the membrane was further probed with antibodies against CDK4 (EPR4513-32-7, abcam, dilution 1:1000) and CDKN2A (G175-405, BD Pharmingen, San Jose, CA, USA, dilution 1:500).  Protein expression of the total retinoblastoma protein (RB) (4H1, Cell Signaling Technology, dilution 1:2000) and its member p130/RBL2 (D9T7M, Cell Signaling Technology, Danvers, MA, 1:1000) were also investigated at 4°C overnight. Signal detection was achieved by incubation with HRP-labeled secondary antibodies goat-anti rabbit (111-035-144) and goat-anti mouse (115-035-003, Jackson ImmunoResearch Laboratories, Cambridgeshire, United Kingdom, dilution 1:10000) at RT for 1 h and Amersham ECL Prime reagent (GE Healthcare) visualizing the protein-antibody complex by enhanced chemiluminescence. The signal was captured on X-ray film (Fujifilm Corporation, Tokyo, Japan). Reprobing with the α-tubulin antibody (DM1A, Sigma-Aldrich 1:20000) was used to normalize protein levels. Autoradiographs were scanned and quantification of individual bands was performed with the FIJI software (version 2.0.0). |

**Supplementary Table 2. Baseline clinical and histopathological data in the entire cohort of patient adrenocortical carcinoma (n=104) stratified by CDK4 protein expression.**

|  | low CDK4 expression  (H-score ≤1) | high CDK4 expression  (H-score >1) | P value |
| --- | --- | --- | --- |
| n | **72** | **32** | **-** |
| Sex (F/M) | 43/29 | 16/16 | NS |
| Baseline |  |  |  |
| Age – yrs (median, range)  < 50 years – *n (%)*  ≥ 50 years – *n (%)* | 46 (18-87)  40 (55.6)  32 (44.4) | 51 (25-81)  14 (43.8)  18 (56.2) | NS |
| Steroid secretion – *n* available  Cortisol – *n (%)*  Other single steroids (androgens, mineralocorticoids, or estrogens) – *n (%)*  Mixed steroids – *n (%)*  Inactive – *n (%)* | 55  14 (19.4)  7 (9.7)  14 (19.4)  20 (27.8) | 23  9 (28.1)  2 (6.3)  7 (21.9)  5 (15.6) | NS |
| Tumor localization  Primary tumor – *n (%)*  Local recurrences – *n (%)*  Metastases – *n (%)* | 57 (79.2)  7 (9.7)  8 (11.1) | 30 (93.8)  1 (3.1)  1 (3.1) | *0.08* |
| ENSAT tumor stage  I-II – *n (%)*  III – *n (%)*  IV – *n (%)* | 38 (52.8)  16 (22.2)  18 (25.0) | 17 (53.1)  11 (34.4)  4 (12.5) | NS |
| Resection status – *n* available  R0 – *n (%)*  RX – *n (%)*  R1 – *n (%)*  R2 – *n (%)* | 70  46 (63.9)  12 (16.7)  5 (6.9)  7 (9.7) | 31  26 (81.3)  4 (12.5)  0 (0)  1 (3.1) | NS |
| Ki67 index – median (range) | 15 (2-90) | 10 (1-80) |  |
| Therapeutic approaches  Additional surgeries – *n (%)*  Radiotherapy (tumor bed or metastases) – *n (%)*  Mitotane  Adjuvant setting – *n (%)*  Palliative setting – *n (%)*  Cytotoxic chemotherapies  None – *n (%)*  Platinum-based regimen – *n (%)*  Streptozotocin – *n (%)*  Gemcitabin plus capecitabin – *n (%)*  Iodmetomidate – *n (%)* | 30 (41.7)  25 (34.7)  28 (38.9)  32 (44.4)  27 (37.5)  38 (52.8)  31 (43.1)  27 (37.5)  4 (5.6) | 8 (25.0)  7 (21.9)  10 (31.3)  6 (18.8)  14 (43.7)  15 (46.9)  12 (37.5)  10 (31.3)  0 (0) | NS  NS  NS  *0.01*  NS  NS |

**Abbreviations:** F=female; M=male; n=number of patients; R0=complete resection; R1=microscopic incomplete resection; R2=macroscopic incomplete resection; RX=uncertain resection; yrs=years.

# Legends to Supplementary Figures

**Supplementary Figure 1. Association between *CDK4* and *IGF1R* gene expression in the sub-cohort of 40 adrenocortical carcinoma (ACC) samples used for mRNA analyses.**

(A) Number of ACC samples with different *CDK4* and *IGF1R* mRNA expression levels. Statistical analysis by Chi-square test.

(B) Relationship between *CDK4* mRNA FC and *IGF1R* mRNA FC (*p=0.25*). The regression line is shown. Statistical analysis by linear regression.

**Supplementary Figure 2. Relative *CDK4* gene expression based on pre-existing data from Affimetrix U133 plus 2 arrays ^25^.** *CDK4* gene expression (quantile normalized and log transformed), in adrenocortical carcinomas (ACC, n=33), adrenocortical adenomas (ACA, n=22) and normal adrenal glands (NAG, n=10). FC=fold changes. Bars represent median and interquartile range. **p<0.01. Statistical analysis by Kruskal-Wallis test.

**Supplementary Figure 3. Protein expression of CDK4-related factors in NCI-H295R and MUC1 cell lines.**

(A) Western blots of CDK4, CDKN2A/p16^INK4A^, RB1 and p130/RBL2 for NCI-H295R and MUC1 cells. The adrenocortical carcinoma cell line NCI-H295 (H295 suspension cells) was additionally investigated for screening purposes. Hek293 and Hela cells were used as positive controls.

(B) Quantitative western blot analysis of CDK4, CDK2NA/p16^INK4A^, RB1 and p130/RBL2 for NCI-H295R and MUC1 cells as well as Hek293 and Hela cells. α-tubulin was used as the internal standard. Each bar of the histograms represents the mean of the ratio of protein of interest to α-tubulin signal (n=1).

**Supplementary Figure 4. CDK4 knockdown in NCI-H295R and MUC1 cell lines.**

(A) Relative *CDK4* mRNA fold change by RT-qPCR 72h after transfection with CDK4 siRNA and compared to cells treated with control siRNA. β-actin was used as the housekeeping gene. Experiments were conducted in triplicates.

(B) Relative CDK4 protein expression by quantitative western blot analysis 72h after transfection. α-tubulin was used as internal standard. Each corresponding control was defined as 1.0, each bar of the histograms represents the relative ratio of CDK4 to α-tubulin signal. Experiments were conducted in triplicates.

(C) Cell viability measured by WST1 test 72h after transfection. The mean of the absorbance measured for cells transfected with control siRNA was defined as 100% for each experiment. The ratio of measured absorbance to the mean absorbance forms the final data. For both cell lines three independent experiments were conducted using octuplet samples.

Statistical analysis by unpaired t-test with Welch‘s correction. ns = p not significant, *p<0.05, **p<0.01, ***p<0.001.

**Supplementary Figure 5. Effects of treatment with palbociclib and linsitinib alone in NCI-H295R and MUC1 cell lines.**

(A) Comparison of cell viability in NCI-H295R and MUC1 cells after palbociclib treatment at 48, 96, 144 and 192h. Cells were treated as described in Figure 4A.

(B) Interpolation of cell viability of NCI-H295R (left) and MUC1 (right) cells after treatment with linsitinib. Cell viability was measured by WST1 test after administration of linsitinib as described in Figure 4. For both cell lines three independent experiments were conducted using octuplet samples. *p<0.05, ***p<0.001 for comparison with the control sample. Statistical analysis by two-way ANOVA.
